# Available evidence on HIFU for focal treatment of prostate cancer: a systematic review

**DOI:** 10.1590/S1677-5538.IBJU.2021.0091

**Published:** 2021-04-20

**Authors:** Arnas Bakavicius, Giancarlo Marra, Petr Macek, Cary Robertson, Andre L. Abreu, Arvin K. George, Bernard Malavaud, Patrick Coloby, Pascal Rischmann, Marco Moschini, Ardeshir R. Rastinehad, Abhinav Sidana, Armando Stabile, Rafael Tourinho-Barbosa, Jean de la Rosette, Hashim Ahmed, Thomas Polascik, Xavier Cathelineau, Rafael Sanchez-Salas

**Affiliations:** 1 Vilnius University Faculty of Medicine Vilnius Lithuania Faculty of Medicine, Vilnius University, Vilnius, Lithuania; 2 Institut Mutualiste Montsouris Department of Urology Paris France Department of Urology, Institut Mutualiste Montsouris, Paris, France; 3 Duke University Department of Urology Durham NC USA Department of Urology, Duke University, Durham, NC, USA; 4 Keck School of Medicine and University of South California Department of Urology CA USA Department of Urology, Keck School of Medicine and University of South California, CA, USA; 5 University of Michigan Department of Urology Ann Arbor MI USA Department of Urology, University of Michigan, Ann Arbor, MI, USA; 6 Institut Universitaire du Cancer Toulouse Oncopole Department of Urology Toulouse France Department of Urology, Institut Universitaire du Cancer Toulouse Oncopole, Toulouse, France; 7 Centre Hospitalier René-Dubos Department of Urology Pontoise France Department of Urology, Centre Hospitalier René-Dubos (Pontoise), France; 8 Vita-Salute San Raffaele University IRCCS San Raffaele Scientific Institute Department of Urology and Division of Experimental Oncology Milan Italy Department of Urology and Division of Experimental Oncology, Vita-Salute San Raffaele University, IRCCS San Raffaele Scientific Institute, Milan, Italy; 9 Lucerne Kanton Hospital Department of Urology Lucerne Switzerland Department of Urology, Lucerne Kanton Hospital, Lucerne, Switzerland; 10 Lenox Hill Urology Department of Urology NY USA Department of Urology, Lenox Hill Urology, NY, USA; 11 University of Cincinnati College of Medicine Division of Urology Cincinnati OH USA Division of Urology, University of Cincinnati College of Medicine, Cincinnati, OH, USA; 12 Faculdade de Medicina do ABC Departamento de Urologia São Paulo Brasil Departamento de Urologia, Faculdade de Medicina do ABC (Faculdade de Medicina do ABC), São Paulo, Brasil; 13 Istanbul Medipol Mega University Hospital Department of Urology Istanbul Turkey Department of Urology, Istanbul Medipol Mega University Hospital, Istanbul, Turkey; 14 Imperial College London Faculty of Medicine Department of Surgery & Cancer London United Kingdom Faculty of Medicine, Department of Surgery & Cancer, Imperial College London, United Kingdom

**Keywords:** Prostate cancer, familial [Supplementary Concept], High-Intensity Focused Ultrasound Ablation, Technology, complications [Subheading]

## Abstract

**Purpose:**

Prostate cancer (PCa) is the second most common oncologic disease among men. Radical treatment with curative intent provides good oncological results for PCa survivors, although definitive therapy is associated with significant number of serious side-effects. In modern-era of medicine tissue-sparing techniques, such as focal HIFU, have been proposed for PCa patients in order to provide cancer control equivalent to the standard-of-care procedures while reducing morbidities and complications. The aim of this systematic review was to summarise the available evidence about focal HIFU therapy as a primary treatment for localized PCa.

**Material and methods:**

We conducted a comprehensive literature review of focal HIFU therapy in the MEDLINE database (PROSPERO: CRD42021235581). Articles published in the English language between 2010 and 2020 with more than 50 patients were included.

**Results:**

Clinically significant in-field recurrence and out-of-field progression were detected to 22% and 29% PCa patients, respectively. Higher ISUP grade group, more positive cores at biopsy and bilateral disease were identified as the main risk factors for disease recurrence. The most common strategy for recurrence management was definitive therapy. Six months after focal HIFU therapy 98% of patients were totally continent and 80% of patients retained sufficient erections for sexual intercourse. The majority of complications presented in the early postoperative period and were classified as low-grade.

**Conclusions:**

This review highlights that focal HIFU therapy appears to be a safe procedure, while short-term cancer control rate is encouraging. Though, second-line treatment or active surveillance seems to be necessary in a significant number of patients.

## INTRODUCTION

Prostate cancer (PCa), with almost 1.3 million new cases and 359.000 deaths annually, is the second-most frequent oncologic disease and the fifth leading cause of cancer-related death among men (1). In the past decade, because of greater public and professional awareness as well as widespread use of molecular testing, PCa diagnosis has shifted dramatically towards earlier-stage, localized disease (2). Radical treatment with curative intent provides good oncologic results for these patients, although definitive therapy is associated with many serious side-effects (3), all of which have a significant negative impact on a man's physical and mental well-being (4). As part of the tremendous progress being made in new treatment modalities, minimally-invasive procedures, such as high-intensity focused ultrasound (HIFU), have been suggested for PCa patients with the intent of providing equivalent oncologic safety to the standard of care with a reduced side-effect profile (5). Targeting energy at a focal lesion in or on the prostate, thereby sparing surrounding, non-cancerous prostate tissue, could reduce treatment-related toxicity to the minimum level possible, thus raising interest in focal HIFU therapy is seen in uro-oncological society (6). In this systematic review, we provide the available evidence about focal HIFU therapy as a primary treatment for treatment-naïve localized PCa. Our objective is to review technical aspects of the procedure, oncologic and functional outcomes, complications, as well as retreatment options.

## MATERIALS AND METHODS

This systematic review was based on recommendations by Cochrane (7) and Preferred Reporting Items for Systematic Reviews and Meta-Analyses (PRISMA) protocols (8). The review protocol was registered in the PROSPERO database (CRD42021235581). Web search was performed on the MEDLINE database (through PubMed) using the key words <prostate cancer> AND <HIFU focal therapy> OR <HIFU partial gland ablation> OR <HIFU focal therapy outcomes> OR <HIFU focal therapy complications> OR <retreatment after HIFU focal therapy>. The search was limited to articles published in the English language and reporting focal HIFU outcomes between 2010 and 2020, including meta-analyses, randomised controlled trials, prospective development studies, prospective and retrospective case series with more than 50 patients. Two authors performed abstract screening independently. In case of discrepancies, uncertainty was solved by a third author. Web search was implemented by manual search including senior authors consultation and search of included articles reference lists. Review articles, case reports, and congress abstracts as well as studies reporting whole-gland ablation or procedures performed in a salvage setting were excluded. The following data were extracted from each study: study design, study population, technical aspects of the procedure, type of anaesthesia, urinary catheter management, length of follow-up, oncologic and functional outcomes, and complications and recurrence management.

## RESULTS

### Evidenced synthesis

**Figure 1:**
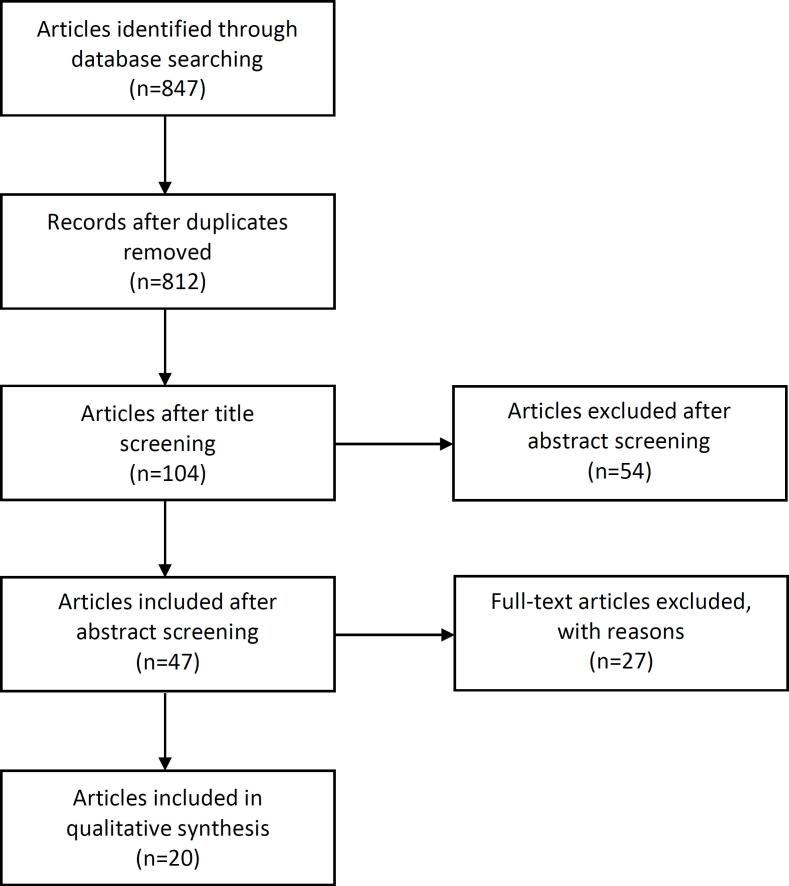
PRISMA flow diagram for articles included into the review.

Of 847 studies identified, we included 20 in the final analysis (Figure-1): 1 randomised controlled trial (9), 10 prospective development studies (10–19), and 9 retrospective case series (6, 20–27). Across all the studies, 4209 patients were treated with focal HIFU. Study design and data extracted from each study are summarised in Table-1.

**Table 1:** Study design, oncological and functional outcomes, as well as complications and retreatment of the 20 studies included.

Author, years	Study design	Patients, N	Prostate cancer risk group*	Type of ablation	Follow-up, months		Cohort with control biopsy,%	In-field recurrence, %		Out-of-field progression, %		Reported functional outcomes	Reported adverse events	Reported retreatment tactics
								Any	CS	Any	CS			
Bakavicius et al. 2019 (6)	Retrospective cases series	210	Low, intermediate	Lesion-targeted ablation, hemiablation, subtotal ablation	11		–	–	–	–	–	–	Yes	–
Hamdy et al., 2018 (9)	Randomized control trial	41	Intermediate	Lesion-targeted ablation, quadrant ablation, hemiablation	–		–	–	–	–	–	–	–	–
Ahmed et al., 2015 (10)	Prospective development study	56	Low, intermediate	Lesion-targeted ablation	12		92.9	34.6	15.4	7.7	3.8	Yes	Yes	Yes
Dickinson et al., 2017 (11)	Prospective development study	118	Low, intermediate	Lesion-targeted ablation, hemiablation	12		94.1	36.9	18.9	–	–	–	–	–
Feijoo et al., 2016 (12)	Prospective development study	67	Low, intermediate	Hemiablation	12		100a	16.4a	–	10.4a	–	Yes	Yes	–
Guillaumier et al., 2018 (13)	Prospective development study	625	Low, intermediate, high	Quadrant ablation, hemiablation	56		35.5	18.0	–	12.2	–	Yes	Yes	Yes
Rischmann et al., 2017 (14)	Prospective development study	111	Low, intermediate	Hemiablation	30		91.8	13.9	5.0	20.8	6.9	Yes	Yes	Yes
van Velthoven et al., 2016 (15)	Prospective development study	50	Low, intermediate	Hemiablation	35		16.0b	6.0b	–	10.0b	–	Yes	Yes	Yes
Schmid et al., 2020 (16)	Prospective development study	98	Low, intermediate	–	3		–	–	–	–	–	–	Yes	–
Lovegrove et al., 2020 (17)	Prospective development study	420	Low, intermediate, high	Lesion-targeted ablation, quadrant ablation, hemiablation, hockey stick ablation, subtotal ablation	65 and 73		–	–	–	–	–	Yes	–	–
Ganzer et al., 2018 (18)	Prospective development study	51	Low, intermediate	Hemiablation	17		96.1	26.5	8.2	34.7	2.0	Yes	Yes	Yes
Mortezavi et al., 2019 (19)	Prospective development study	75	Low, intermediate	–	6		90.7	–	20.6	–	29.4	Yes	–	Yes
Albisinni et al., 2017 (20)	Retrospective cases series	55	Low, intermediate	Hemiablation	36		–	12.7b	9.1b	21.8b	–	Yes	Yes	Yes
Tourinho-Barbosa et al., 2020 (21)	Retrospective cases series	190	Low, intermediate	Lesion-targeted ablation, quadrant ablation, hemiablation, subtotal ablation	37		91.6	30.0	–	16.8	–	Yes	Yes	Yes
Stabile et al., 2019 (22)	Retrospective cases series	1032	Low, intermediate	Lesion-targeted ablation, hemiablation	36		41.1	31.5				–	-–	Yes
Johnston et al., 2019 (23)	Retrospective cases series	107	Low, intermediate	Lesion-targeted ablation, quadrant ablation, hemiablation	30		62.6	28.0				Yes	Yes	Yes
Bass et al., 2019 (24)	Retrospective cases series	150	Low, intermediate, high	Lesion-targeted ablation, hemiablation, hockey stick ablation	24		58.0	–	12.7	–	12.0	Yes	Yes	Yes
Annoot et al., 2019 (25)	Retrospective cases series	55	Low, intermediate	Hemiablation	33		–	–	21.8	–	5.5	–	–	Yes
Huber et al., 2020 (26)	Retrospective cases series	598	Low, intermediate, high	Lesion-targeted ablation, quadrant ablation, hemiablation	–		–	35.1c				–	–	–
Abreu et al., 2020 (27)	Retrospective cases series	100	Low, intermediate, high	Hemiablation	18		58.0	10.0	8.0	23.0	10.0	Yes	Yes	Yes

* Prostate cancer risk stratification is based on modified D'Amico classification system according to EAU Prostate Cancer guidelines 2020. The grouping reflects the biggest part of the cohort included into the study.

For visual purpose “-” = symbol was used when specific data was not available in a study. In some studies control biopsies were performed not routinely per-protocol and (a) based on scheduled clinical visits without post-operative mpMRI, (b) on PSA kinetics only, as well as (c) triggered only when a suspicious lesion on post-operative mpMRI was detected or PSA rising was observed. Abbreviations: CS - clinically significant; EAU - European Association of Urology; mpMRI - multiparametric magnetic resonance imaging; N - number of patients; PSA - prostate-specific antigen.

### Technical aspects of the procedure

All the patients underwent transrectal focal HIFU using three devices: Sonablate 500 (SonaCare Medical LLC; 8 studies and 2786 patients) (10, 11, 13, 22–24, 26, 27), Ablatherm Fusion (EDAP TMS; 8 studies and 778 patients) (6, 12, 15, 18, 20, 21, 25, 27), and Focal One (EDAP TMS; 6 studies and 679 patients) (6, 16, 18, 19, 21, 25). The procedure was performed with the patient in the lateral or supine position under general (9 studies) (6, 9, 10, 14, 15, 18, 19, 23, 27) or spinal anaesthesia (4 studies) (9, 14, 15, 24). Four authors used a 6-mm safety margin from the apex of the prostate to preserve sphincter functionality and maintain continence (6, 12, 15, 25), while in one study (24) the safety distance was decreased to 3mm and in another one (16) it was increased to 10mm.

Bladder catheterization with an indwelling urethral catheter (11 studies) (6, 9, 12, 14–16, 18, 22, 24, 25, 27) or suprapubic approach (5 studies) (9, 10, 16, 22, 27) was maintained for 1 to 14 days postoperatively (6, 9, 10, 16, 18, 20, 22, 23, 25, 27). Four of 11 studies with a urethral catheter discontinued bladder drainage 1 to 3 days postoperatively (6, 15, 18, 25), while in another 4 studies, the bladder catheter was removed one week after the procedure (9, 16, 22, 27).

Antibiotic prophylaxis ranged from a single dose intraoperatively (2 studies) (6, 16) up to 7 days after the surgery, usually until the bladder catheter was removed (3 studies) (22, 23, 27). Transurethral resection of the prostate (TURP) was proposed for men with a prostate size greater than 50cm3 (6, 14, 21, 27) or lower urinary tract symptoms (6, 14, 16, 25). TURP was performed in 6 of 20 studies (6, 14, 15, 21, 25, 27), while α-blocker therapy was reported just by 1 author (6). TURP was typically performed within 6 to 12 months preoperatively (14, 16). Few authors considered concomitant TURP with focal therapy under the same anaesthesia (14, 15), while van Velthoven et al. (15) reported a modified version of TURP (including just 1 treated lobe of the prostate) at the end of focal therapy. Two authors (9, 16) considered TURP as an exclusion criterion if it was performed 6 months before the surgery. In 2 studies (17, 26), androgen-deprivation therapy (ADT) was initiated in a subset of patients to reduce prostate volume, while another 4 authors (6, 16, 18, 21) considered ADT as an exclusion criterion.

### Oncologic outcomes

Oncologic outcomes were reported in 16 of 20 studies (10–15, 18–27) included in this review. Median follow-up ranged from 6 to 56 months; the studies with the longest follow-up periods were Tourinho-Barbosa et al. (21) at 37 months and Guillaumier et al. (13) at 56 months.

Median time to reach prostate-specific antigen (PSA) nadir varied from 3 to 12 months postoperatively (10, 14, 15, 21, 24, 27), with the median PSA reduction being 53% to 84% (10, 11, 14, 15, 18, 19, 21, 23, 24, 27). During follow-up, 11 of 16 studies (10, 11, 13, 14, 18, 19, 21, 24–27) reporting oncological outcomes performed multiparametric magnetic resonance imaging (mpMRI) 6 to 12 months postoperatively or at any time earlier if it was clinically indicated, followed by control biopsies afterwards. Three studies have performed follow-up sampling without postoperative mpMRI, based on the PSA kinetics only (15, 20) or scheduled clinical visits (12). According to the literature, including articles only with control mpMRI followed by targeted and systematic biopsies, clinically significant in-field recurrence and out-of-field progression were detected in 5% to 22% (10, 11, 13, 14, 18, 19, 24, 25, 27) and 2% to 29% (10, 14, 18, 19, 24, 25, 27) patients with PCa, respectively. Any type of PCa in the field was detected in 10% to 37% of patients (10, 11, 13, 14, 18, 21, 27), while out-of-field PCa was detected in 8% to 35% of patients (10, 13, 14, 18, 21, 27). Higher International Society of Urological Pathology (ISUP) grade group (21), more positive cores (24), bilateral PCa at primary biopsy (24, 27), and higher postoperative PSA nadir (11, 21, 24, 25) have been identified as the main predictors of disease recurrence. Oncologic outcomes from the first randomised controlled trial (9) are waited in the near future.

### Functional outcomes

Thirteen of 20 studies (10, 12–15, 17–21, 23, 24, 27) have reported functional outcomes after focal HIFU therapy. Urinary and erectile function, as well as health-related quality of life (QOL) have been evaluated by self-reported symptoms and various questionnaires, including the International Prostate Symptom Score (IPSS) (28) - 9 studies (10, 12–14, 17–19, 21, 27), International Continence Society (ICS) male short-form questionnaire (ICSmaleSF) (29) - 3 studies (12, 18, 21), International Index of Erectile Function (IIEF) questionnaire (30) - 6 studies (10, 12, 14, 18, 19, 27), Expanded Prostate Cancer Index Composite (EPIC) questionnaire (31) - 4 studies (10, 13, 17, 19) and Functional Assessment of Cancer Therapy-Prostate (FACT-P) questionnaire (32) - 2 studies (10, 19). Incontinence was defined as the use of any pad in 9 studies (10, 12, 14, 15, 17, 20, 21, 23, 27) or more than 1 pad per day in 2 studies (13, 19). Erectile dysfunction (ED) was defined as the persistent inability to attain and maintain an erection sufficient to permit satisfactory sexual intercourse (19).

At 3 and 6 months after focal HIFU therapy, 86% to 98% (15, 20, 21) and 90% to 98% (19, 20) of patients with PCa self-reported as totally continent, while 12 months after the procedure, complete continence was achieved in 93% to 97% of patients (10, 14, 15, 17, 20, 24). No further improvement in urinary function was observed 2 to 3 years after the procedure (17). IPSS score remained unchanged during the first 6 months postoperatively (12, 18, 19). Interestingly, Abreu et al. (27) and Rischmann et al. (14) reported improved initial IPSS results after focal therapy, therefore, it should be highlighted that preoperative TURP in these studies was performed according to the study protocol. On the EPIC urinary domain questionnaire, the incontinence score showed initial deterioration, although 6 months after the procedure, the score had returned to baseline (19) and remained high at 2 (97% continent) and 3 years (98% continent) afterwards (13). No changes in ICSmaleSF score were detected for 85% of patients with PCa 3 months after the procedure (21), although the same improvement from baseline was observed 12 months after focal therapy (18).

According to self-reported symptoms for erectile function, 69% to 80% of patients with PCa had retained sufficient erections for sexual intercourse 6 months after focal HIFU (19, 20), and these rates remained stable (14, 15, 17, 20, 24) or improved slightly (86%) (23) within the next 2 years. The total score on the 15-question IIEF questionnaire (IIEF-15) initially decreased by 23 points, with a gradual recovery during the early postoperative phase, 6 months after the procedure, the total score was still inferior by 17 points compared with baseline (19). Another study (10) evaluated erectile function in the later postoperative period (12 months) using the same IIEF-15 questionnaire, where 88% of patients reported normal erectile function. A few authors used the IIEF short-form questionnaire (IIEF-5) after focal HIFU. Abreu et al. (27) reported no deterioration in erectile function, while Feijoo et al. (12) and Ganzer et al. (18) reported that 52% to 70% of patients with PCa retained the same preoperative values on the IIEF-5 after the procedure. Just one study (17) reported ED rates after a second focal HIFU, where retreatment was associated with a 7% increased ED rate.

No negative side-effects on bowel function were detected on the EPIC bowel domain questionnaire (19), and no deterioration in QOL was registered on the FACT-P questionnaire (10, 19).

### Complications

Complications after focal HIFU have been reported in 13 of 20 studies (6, 10, 12–16, 18, 20, 21, 23, 24, 27). Overall, 13% to 41% patients with PCa undergoing focal HIFU experienced some type of complication (6, 12–16, 18, 20, 24, 27). The most common treatment-related side effects were acute urinary retention (7% - 27% of patients) (6, 14–16, 18, 20, 24, 27), urethral sloughing (7% - 43%) (6, 10, 14, 27), and urinary tract infection (UTI) (5% - 18%) (6, 10, 13–16, 18, 27), followed by acute infective epididymitis (2% - 8%) (6, 13), fistula (0.3% - 3%) (13, 24), and iatrogenic urethral stricture disease (2% - 4%) (6, 15, 23).

The majority of complications (85% - 100%) presented in the early postoperative period - that is, up to 3 months after the procedure (6, 16, 27). In terms of severity, 80% to 100% of complications were classified as minor (Clavien-Dindo grade I-II), not requiring any surgical intervention (6, 10, 12, 15, 16, 18, 20, 24, 27). The majority of cases of acute urinary retention were managed conservatively with temporary bladder catheterization or α-blocker therapy (6, 12, 16, 24, 27). The most commonly reported interventions for treating grade III to V complications were suprapubic bladder catheterization, TURP, urethrotomy, urethroplasty, and surgical management of fistula (6, 12, 13, 15, 16, 18, 24).

Ablation volume (6) and inclusion of the urethra (16) were identified as the main predictors for postoperative complications. The majority of the patients (78%) undergoing subtotal HIFU reported having some type of complication, while more precise, lesion-targeted ablation was associated with a significantly lower (36%) risk of side-effects (6). Inclusion of the urethra in the HIFU ablation zone led to adverse events (AEs) in 48.8% of patients, while patients undergoing urethra-sparing surgery had a significantly lower (26.3%) risk of complications (16). Other risk factors included smaller prostate volume, higher body mass index, and longer bladder catheterization time (6).

### Recurrence management

Recurrence management was reported in 13 of the 20 studies (10, 13–15, 18–25, 27) included in this review. A second focal HIFU ablation procedure to treat in-field recurrence or out-of-field progression after 1 focal HIFU therapy session was reported in 2 (14, 23) and 4 (14, 15, 20, 23) studies, respectively, where 5% to 11% and 2% to 13% of patients with PCa, respectively, undergoing focal HIFU ablation were retreated in this manner. According to other authors, 4% to 19% of patients with PCa underwent a second focal HIFU ablation procedure, although these authors did not specify whether in-field or out-of-field disease was treated (10, 13, 19, 21, 22, 24, 27).

Other retreatment strategies after one focal HIFU ablation included focal cryotherapy, salvage whole-gland therapies, and ADT. According to Stabile et al. (22), salvage focal cryotherapy was performed in 1% of patients. Salvage whole-gland therapies consisted of whole-gland HIFU, radical prostatectomy (RP), and external beam radiation therapy (EBRT). Salvage whole-gland HIFU ablation was performed in 0.4% to 10% of patients with PCa (14, 18, 22), salvage RP was performed in 1% to 22% of patients with PCa (13, 14, 18–20, 22–25, 27), and salvage EBRT with or without ADT was performed in 0.9% to 8% of patients (10, 13–15, 18, 20, 22–24). A small number of patients (0.2% - 6%) were treated with ADT only, usually because of metastatic disease at the time of recurrence (13, 15, 20, 22–24).

Some patients with low-risk disease, mostly those harbouring ISUP grade group 1 PCa, were offered deferred treatment options. According to 5 articles (10, 14, 18, 19, 25), 4% to 35% of patients with PCa were under active surveillance after focal HIFU therapy.

Two studies (13, 14) reported third-line salvage treatment after 2 focal HIFU ablation procedures, including third-line focal HIFU ablation, which was performed in 1% of patients (13), salvage RP, which was performed in 1% of patients (14), salvage EBRT, which was performed in 2% of patients (14), and ADT only, which was used in 1% of patients (14). A second focal HIFU therapy procedure was associated with increased risk (by 7%) of ED (17) but did not compromise continence (21), no serious AEs were detected after any type of salvage therapy (21, 24).

## DISCUSSION

Since the introduction of aggressive PSA testing into clinical practice, the diagnosis of PCa has shifted dramatically towards earlier-stage and localized disease (2). In the past decade, because of the widespread use of mpMRI and rapid progress in ultrasound and mpMRI-ultrasound fusion technologies, the more precise detection of early-stage PCa has moved towards early-stage, clinically significant disease, which carries the highest risk of progression (33). Taking into account that active treatment is crucial for this type of PCa and that whole-gland therapies are associated with serious AEs in the sexual and urinary domains (3, 4), focal therapy could be a better option for these patients.

Focal HIFU focuses ultrasound waves at a malignant lesion in or on the prostate, it produces heat above 65°C and destroys the targeted area through coagulative necrosis. Since the early 2000s, 3 HIFU devices have been commercially available for PCa treatment: Sonablate 500, Ablatherm, and Focal One. All three machines have been used in some of the studies included in this review: the Sonablate 500 device in 8 studies, the Ablatherm device in 8 studies, and the Focal One device in 6 studies. Some differences in technical characteristics exist between these devices, what could also have affected treatment outcomes (34). The Ablatherm and Focal One device use coupling liquid to protect the rectum; thus, patients should be placed in a lateral position to allow gas bubbles to rise outside the imaging and therapy fields. The Sonablate 500 device uses circulating chilled water instead of coupling liquid, thus, a supine patient position is recommended. The Focal One device, which features the latest technology, enables more precise pre-treatment planning and shape and size modifications during the procedure (which are not possible with the Ablatherm device) as well as end-of-treatment validation through contrast-enhanced ultrasound (which is not possible with the Sonablate 500 device) (35).

Across all the studies included in this review, tissue-preserving strategies during focal HIFU therapy were inconsistent. Based on the volume of the disease and the extension within the prostate, different treatment strategies have been proposed: index-lesion or focal ablation, quadrant ablation, hemiablation, and subtotal or hockey-stick ablation (36). After the procedure, the type and length of bladder catheterisation varied significantly among the initial studies. According to some authors (22), a suprapubic approach was based on their personal experience with whole-gland therapy and prolonged voiding problems afterwards. Thus, urethral catheterization was avoided to decrease the risk for urethral strictures. When it became clear that most men re-established voiding less than a week after the procedure, indwelling urethral catheterization for up to 3 days postoperatively was adopted as the standard approach in most of the studies. A few authors have also performed TURP in preoperative or perioperative settings to decrease postoperative sloughing and prolonged need for bladder catheterization. None of these authors has evaluated the impact of TURP on postoperative functional outcomes.

Short-term oncologic results have been reported in the majority of the studies reviewed, while just 2 studies (13, 21) have reached a postoperative follow-up adequate for intermediate-term results. Taking into account the prolonged natural history of PCa and the lack of reliable comparative data on medium- and long-term oncologic outcomes, no final decision could be made regarding cancer control after focal HIFU therapy. PSA nadir in all the studies reviewed had been reached 12 months postoperatively, with a median reduction greater than 50% from baseline. Despite one author (20) using PSA kinetics to trigger postoperative biopsies, it should be highlighted that currently no accepted definition of biochemical disease control following focal therapy has been established, thus, postoperative follow-up could not be based on Phoenix (37), Stuttgart (38), or any other criteria, and postoperative mpMRI with control biopsies are mandatory (39). During short-term follow-up, half of the patients were histologically confirmed as having clinically significant (ISUP grade group ≥2) disease, where in-field and out-of-field PCa was detected in up to 22% and 29% of patients, respectively.

Retreatment possibilities have been reported in some of the studies. Therefore, no data regarding indications for different retreatment strategies, including second focal HIFU or other focal therapies, or for whole-gland treatment options were reported in any of the studies. For low-risk recurrence, a deferred treatment option was offered in the majority of the studies, where up to 35% of patients with PCa after focal HIFU were undergoing active surveillance. The majority of the studies used radical treatment options, such as salvage RP and salvage EBRT, to treat PCa recurrence or progression. Second focal HIFU was initiated in up to 20% of patients with PCa, where in-field recurrence was treated in 11% of patients and out-of-field progression in 13% of patients.

Regarding the overall complication rate, focal HIFU therapy appears to be a threatening procedure in which up to 41% of patients experience some type of complication. It should be noted, however, that the majority of complications are low grade and do not require surgical intervention. The most common side-effects are temporary acute urinary retention, urethral sloughing, and UTI. One author (24) reported a slightly higher number (3%) of postoperative fistulas, but the problem was solved by increased cooling time between treatment pulses in the midzone of the prostate. Regarding self-reported symptoms and various questionnaires, initial deterioration in the urinary domain during the early postoperative phase was observed in 14% of patients with PCa. During the next few months after the procedure, significant improvement in continence rates has been observed, and 1 year after the procedure, only a low percentage of patients presented incontinence of any grade. Regarding erectile function, 70% of patients with PCa reported no changes in early the postoperative period of focal HIFU. Gradual recovery during the early postoperative phase was also observed, and 86% of patients retained sufficient erection for sexual intercourse following the procedure.

## CONCLUSIONS

Focal HIFU therapy appears to be a safe procedure with low-grade complications and to offer good preservation of urinary and erectile function. Its short-term cancer control rate is encouraging, although second-line treatment or active surveillance seems to be necessary in a significant number of patients with PCa. If intermediate- and long-term oncologic outcomes are verified against standard-of-care procedures in high-quality comparative trials, focal HIFU could become the standard of care for well-selected patients with PCa.

## ABBREVIATIONS

ADT: androgen-deprivation therapy
AE: adverse event
EBRT: external beam radiation therapy
ED: erectile dysfunction
EPIC: Expanded Prostate Cancer Index Composite
FACT-P: Functional Assessment of Cancer Therapy-Prostate
HIFU: high-intensity focused ultrasound
ICS: International Continence Society
ICSmaleSF: ICS male short-form questionnaire
IIEF: International Index of Erectile Function
IPSS: International Prostate Symptom Score
ISUP: International Society of Urological Pathology
mpMRI: multiparametric magnetic resonance imaging
PCa: prostate cancer
PSA: prostate-specific antigen
QOL: quality of life
RP: radical prostatectomy
TURP: transurethral resection of the prostate
UTI: urinary tract infection
